# Maternal Satisfaction with Antenatal Care and Associated Factors among Pregnant Women in Hossana Town

**DOI:** 10.1155/2020/2156347

**Published:** 2020-07-28

**Authors:** Dagmawit Birhanu Kebede, Yeshitila Belay Belachew, Desta Workneh Selbana, Admasu Belay Gizaw

**Affiliations:** Jimma University School of Nursing and Midwifery, P.O. Box 378, Ethiopia

## Abstract

**Background:**

A woman's satisfaction with antenatal care service has immediate and long-term impacts on maternal and her baby's health. It also ensures further use of service. However, it is not well studied in Ethiopia in general and at the southern region in particular.

**Objective:**

The main objective of this study is to assess the level of a maternal satisfaction with antenatal care services and associated factors.

**Methods:**

An institution-based cross-sectional study that involves both quantitative and qualitative methods of data collection was employed. A systematic sampling technique was used to obtain study participants, and quantitative data were collected using an interviewer-administered questionnaire. For qualitative data, Focus Group Discussions were done among clients that have a repeated visit by taking educational status as homogeneity criteria. EpiData version 3.1 and SPSS version 21 were used for analysis. Descriptive statistics, bivariate and multivariable logistic regression analyses were employed to describe and identify factors associated with maternal satisfaction on antenatal care. The qualitative data were analyzed thematically and manually.

**Results:**

Overall, 74% of mothers were satisfied with antenatal care services rendered in public health institutions of Hossana town. Most of the respondents were satisfied with privacy, cleanness, physical facility, and approaches of care. Age, educational status, privacy, cleanness, distance, and respect were significantly associated with a client's satisfaction.

**Conclusion:**

Three-fourths of the respondents were satisfied with the service. Age, education, living distance, maintenance of privacy, cleanness of the facility, and respect from providers were the significant predictors of the satisfaction level.

## 1. Introduction

Antenatal care can be broadly defined as encompassing pregnancy-related services provided between conception and the onset of labor with the aim of improving the pregnancy outcome and the heath of the mother or child and providing women and their families/partners with appropriate information and advice for a healthy pregnancy, childbirth, and postnatal recovery [1]. The measurement of patients' satisfaction is a common component of evaluations in quality care. Clients' reported levels of satisfaction reflect professionals' technical competence as judged by independent assessors and are an important indicator of the quality of care. It is known that a satisfied client is more likely to comply with treatment and advice she receives from health care professionals [2]. Client satisfaction was a function of patients' subjective responses to experienced care mediated by personal preferences and expectations. Also, it is a measurement designed to obtain reports or ratings from patients about services received from an organization, hospital, or health care provider. On a practical level, client satisfaction is equivalent to the actual measure [3].

Many maternal and prenatal deaths occur in women with low utilization of antenatal care (ANC), but utilization of ANC service is affected by a client's satisfaction. Nevertheless, true progress has been made globally in terms of increasing access. Worldwide, 70% of women ever receive any antenatal care, whereas in industrialized countries, more than 95% of pregnant women receive ANC [4]. Globally, an estimated 295,000 maternal deaths occurred in 2017, yielding an overall 211 maternal deaths per 100,000 live births [5]. Ninety-nine percent of this death occurs in developing countries, and the sub-Saharan African region alone accounts for 66% (19600) of maternal death. Maternal mortality is higher in women without skilled care before, during, and after childbirth. Ethiopia is one of the countries in sub-Saharan Africa with a markedly high maternal mortality ratio (401 deaths per 100,000 live births) [5].

ANC can reduce maternal mortality by 20% given good quality and regular attendance. ANC attendance during pregnancy has a positive impact on the use of postnatal healthcare services and an important entry point to convince expectant mothers about the health benefits of attended delivery [6–8]. However, the use of ANC service by pregnant women could be affected by the level of their satisfaction with the service provided at the health care facility. In southwestern Nigeria, only 44% of respondents utilized health care facilities. This was attributed to various factors causing dissatisfaction with services rendered at these centers [9–16].

Patients' use of health care is affected by the quality of care; those who are not satisfied with their providers may be less likely to continue with treatment or seek further services. According to the U.S. Agency for Healthcare Research and Quality, National Consumer Assessment of Healthcare Providers and Systems (CAHPS) Benchmarking Database in Africa, a maternal satisfaction survey was conducted at different times in different counties (for instance, in 2012 and 2018 and in Egypt and Nigeria, the level of maternal satisfaction with ANC was greater than 90% and 90%, respectively, whereas in Ethiopia, the levels of maternal satisfaction with antenatal care in 2008, 2013, 2014, 2018, and 2019 were 35%, 47.7%, 60.4%, 90%, and 90.8%, respectively [17–23].

The degree of patient satisfaction can be used as a means of assessing the quality of health care and personnel. It reflects the ability of the provider to meet the patients' needs. Satisfied patients are more likely than the unsatisfied ones to continue using health care services, maintain their relationships with specific health care providers, and comply with the care regimens [24–26]. Sociodemographic background of the patient, expectations of care, organizational factors (facility-related factors), and provider-related factors (communication and information, participation and involvement, and interpersonal relationships) are very important aspects or factors on which patient satisfaction depends [14, 18, 20, 27, 28].

Different studies in different parts of the world revealed that maternal satisfaction with antenatal care was affected by examination room cleanness, health care provider attitude, quality of antenatal care service provided, adequacy of information provided by the health care professionals, waiting time, supervision of antenatal care, adequacy of water supply, adequacy of waiting area, educational status of the mother, monthly income, type of pregnancy and history of stillbirth, patient's previous experiences, social and cultural norms, physical environment, availability of adequate resources (human, medicines, drugs, equipment, and supplies), adequacy of clinical care, and access to treatment in healthcare facilities [14, 29–38].

Despite these facts, client satisfaction on antenatal care service is one of the measures of quality care and is essential for further improvement of maternal and child health, and it is not well studied in the southern region of Ethiopia. Therefore, this study is aimed at assessing the level of maternal satisfaction with antenatal services and its associated factors in public health facilities of Hossana town.

## 2. Materials and Methods

### 2.1. Study Setting

The study was conducted in Southern Nations, Nationality, and People's Region (SNNPR) state, Hadiya zone, Hossana town, in Ethiopia. It is one of the political and administrative towns and lies 230 km southwest of the capital city, Addis Ababa. The town is divided into three subcities and eight kebeles. The total population size of the town projected to the year 2013 according to the 2007 population and housing census was 102,238 of which 50,094 are male and 52,144 are female with an annual growth rate of 5%. The census revealed that the town has a sex ratio of 51 : 49 females to males. The population of women in the reproductive age (WIRA) was 23,821, and the proportion of pregnant women constitutes 3.9% of the total population. Currently, in 2019, the town has one hospital, three health centers, and sixteen private clinics.

### 2.2. Study Design

A facility-based cross-sectional study that involves both qualitative and quantitative methods was conducted.

### 2.3. Sample Size Determination

The sample size for the quantitative study was determined using a single population proportion formula, and a total of 325 pregnant women are recruited:

(1) $$\begin{array}{c} n=\frac{\left(Z\alpha /2\right)2xp\left(1-p\right)}{d^{2}}, \end{array}$$

where *n* is the sample size; *Zα*/2 is the confidence level at 95% = 1.96; *P* is the proportion of satisfaction of women which is unknown, assumption of 50% (*P* = 0.5); and *d* is the margin of error of 4.5%:

(2) $$\begin{array}{c} n=\frac{{\left(Z\alpha /2\right)}^{2}*P\left(1-P\right)}{d^{2}},\\ n=\frac{{\left(1.96\right)}^{2}*0.5\left(1-0.5\right)}{{\left(0.05\right)}^{2}},\\ n=384. \end{array}$$

Since the source population is less than 10,000, the correction formula was used to get the final sample size as follows:

(3) $$\begin{array}{c} \text{nf}=\frac{n}{1+n/N}, \end{array}$$

where '*N*' is the source population-all estimated pregnant women, 'nf' is the required sample size, and *n* is the calculated sample size. Hence, the sample size will be calculated at a total of source population *N* = 1280, *n* = 384, and *nf* = 295. Considering the nonresponse rate of 10%, the total sample size will be 325 pregnant women.

For the qualitative study, 32 pregnant women on an ANC follow-up were recruited out of the quantitative study area and categorized into 4 focus group discussions (FGDs) of equal size.

### 2.4. Sampling Technique

The entire four public health facilities in the town were included in the sampling process. The calculated sample size was proportionately allocated for each health facility, and the selection was performed using a systematic sampling technique. The number of clients in each health facility was determined by reviewing the annual document of each health facility and the number of subjects to be included in the study from each health facility. The first participant was randomly selected using a lottery method on the first date, and then, every fourth ANC client was selected.

### 2.5. Data Collection Instrument and Data Collection Procedure

Data was collected by a structured questionnaire. The questionnaire was adapted by reviewing relevant literatures on measures of satisfaction and modified to the local context (PSQ 18, PSQ-46, PSQ II SERVQUAL). The questionnaire consists of 27 items which measure the level of satisfaction with the different factors that contribute to client satisfaction of ANC services, i.e., provider-related factors and facility-related factors, sociodemographic variables, and clients' degree of knowledge, and service expectation of the respondents before utilizing the institution service was also included in the questionnaire. The questionnaire was first developed in English for the interview with pregnant women then translated first into Amharic and Hadiyesa and back-translated to English by someone who was native to those local languages to ensure its consistency and to make it simple during administration. The questionnaire was pretested on 5% of the sample in Balesa town before the data collection period to ensure clarity of the questionnaires, and then, necessary modifications and correction were made to ensure its validity. The reliability (internal consistency of the instrument) was cheeked by Cronbach's alpha. It measures the reliability of the instrument between each domain and the whole of the instrument. All domains had Cronbach's alpha greater than 0.7. The questionnaire consisted of five-point Likert scale items, with 1 and 5 indicating the lowest and highest levels of satisfaction, respectively. The level of satisfaction was indicated by selecting responses ranging from 5 = strongly agree, 4 = agree, 3 = uncertain, and 2 = disagree to 1 = strongly disagree. Five clinical nurse data collectors were selected from the health institution with two supervisors.

They were oriented for a day by the principal investigator about the study instrument, consent form, how to interview, and data collection procedure. Qualitative data focus group discussion (FGD) was conducted among clients that did not participate in the quantitative study in the same time period. Four FGDs were carried out, in the selected two public health facilities by taking educational status as homogeneity criteria. Each group had 6-10 members, an average of 8 female ANC users. The FGD was mediated by the principal investigator; the discussion of the participants was both audiotaped and manually written by two other note-takers and analyzed thematically and manually.

### 2.6. Data Quality Control

Data were collected by trained data collectors, and pretesting of the instrument was made out of the study site before the actual data collection. Every day, questionnaires were reviewed and checked for its completeness by the selected and trained supervisors and principal investigator; then, the necessary feedback was offered to data collectors in the next morning before data collection. Data were checked on the spot, errors were rectified, and missing data were incorporated into a form. The researcher with the supervisor checked the data collection procedure and counterchecked the entries at random to ensure the quality of data collection.

### 2.7. Ethical Consideration

Ethical clearance was obtained from the Institutional Review Board of Jimma University. A formal letter from the Institute of Health Science was given to the Hadiya zone health bureau and other concerned bodies to obtain their cooperation. An explanatory letter was added to each questionnaire to maintain participant rights; also, all patients were asked to participate in the study and received full explanations about the research purposes; respect, anonymity, and confidentiality were given and maintained by a consent form for each participant; they have the liberty to withdraw at any stage of the interview; and their participation will not undergo any pressure.

## 3. Data Processing and Analysis

Data were analyzed by using SPSS version 21.0 statistical software. The normality of data was tested using the Kolmogorov-Smirnov test. All the quantitative data were found to be not normally distributed if (*p* ≤ .0001). Descriptive statistics was used for determining the frequency, percentage, mean, and standard deviation. Multivariable logistic regression analyses were used to understand the significance of the association between dependent and independent variables. All independent variables with *P* value < 0.05 were considered as statistically significant in this study.

## 4. Results

### 4.1. Sociodemographic Characteristics of Respondents

Among 325 pregnant women attenuating ANC, 303 participated in the study which makes a 93% response rate and four FGDs consisting of 6-8 members. Of the 303 women, 40.3% were below 24 years of age, 37.3% were between 24 and 29 years, and the rest are above 30 years of age (22.5%), and the mean age of the exit interview was 25 years. Around two-thirds of the study subjects (64.7%) were protestant followed by orthodox 21.8%, and the majority (98%) of the women were currently married. Regarding educational status, only 35 (11.6%) were illiterate, 28% were primary school level, and the rest 64.4% were secondary school and above. Regarding respondent ethnicity, more than two-thirds (69%) of them were Hadiya followed by 8.6% Amhara and 7.3% Kembata. Concerning the employment status of the study participants, more than half of the study subjects (56.1%) were unemployed. More than eighty-eight percent of the respondents live in urban whereas the rest (about 12%) live in rural (Table 1).

### 4.2. Level of Client Satisfaction with Different Domains of Satisfaction

Most of the study subjects were satisfied by privacy (91.4%), cleanness of the center (87.5%), facility-related factors (82.8%), approaches of care by the care provider (80.2%), interpersonal and technical aspects (79.9%), and communication and information (75.2%). Overall, more than 74% of the study subjects were satisfied with the service that they received while the others (25.4%) are not satisfied (Table 2).

### 4.3. Factors Associated with Antenatal Care Satisfaction

Age is statistically significantly related to the level of satisfaction (*AOR* = 5.584; 95% CI: 1.76, 17.7). Younger mothers were more likely to be satisfied than their counterparts. Those study participants who are illiterate were four times more likely to be satisfied than those who were in primary school and above (*AOR* = 4.53; 95% CI: 1.228, 16.751). Study subjects whose privacies were maintained were more likely to be satisfied than those whose privacy was not maintained (*AOR* = 8; 95% CI: 3.375, 19.263). Cleanness of the institution is also an independent predictor of satisfaction; those who feel that the institution is clean were more likely to be satisfied than those who said that the institution is not clean (*AOR* = 3.596; 95% CI: 1.424, 9.079).

Distance from home to facility is significantly associated with satisfaction; those who travel less than 30 minutes to reach facilities were two times more likely to be satisfied than those who travel greater than 30 minutes (*AOR* = 2.804; 95% CI: 1.423, 5.522); also, one woman supports this idea from the qualitative finding by saying that “…. In the previous time I did not come to hospital when I need care because I travel long distance to reach health institution, but now the institution is near to my home so that I always came whenever I am in need.” Respect has also a statistically significant association with satisfaction; those who reported that care providers were respectful were more satisfied than those who reported that care providers were not respectful (*AOR* = 2.578; 95% CI: 1.249, 5.325) (Table 3); also, one participant illustrates from qualitative findings: “…. I would say that she (the care provider) is a positive one because she greets me with a smile, and again, warm greets, even if she is exhausted too many clients, but she doesn't show any bored facial expression for me” (Table 3).

## 5. Discussion

Antenatal care (ANC) is the key entry point of a pregnant woman to receive a broad range of health promotion and preventive services which provide the health of the mother and the baby [9]. Quality of ANC is an important determinant of the pregnancy outcome along with clean and safe delivery, essential obstetric care, and family planning which could contribute to the reduction of maternal mortality [10]. The majority of respondents (87.8%) reported that care providers were respectful. This finding is supported by a qualitative finding as one participant said that “I could say that she (the care provider) is a positive one because she greets me with a smile, warm greets, even if she's really tired of too many clients she doesn't act like she was tired.”

Around 70% of the respondents took less than 30 minutes to reach the health facility. This was supported by the qualitative finding as one participant said that “it only took me around ten minutes to reach to the health institution it is very important that facilities were near to my home if I get seek or in need of immediate care it will be easy to reach.” This finding is lower than that of a study conducted in Pakistan and Nepal which declared that it was easily accessible (within 30 minutes) [38]. A possible explanation for this might be the difference in infrastructure and economical difference in the countries [11]. Overall, in this study, almost 75% of women were satisfied with the antenatal care service rendered in the health institutions and 25.4% were dissatisfied. This finding is similar to a study conducted in Jimma University Specialized Hospital where 77% of respondents were satisfied [28]. This finding is not consistent with a study conducted in Belgium, at the University Hospital of Ghent (Belgium), Australia, Sweden, Nigeria, Shire North West Tigray, Bahir Dar, and Jimma town mothers in which majority of the study participants were satisfied by antenatal care services [13–20]. The difference might be due to competency, attitude, and skills of maternal care providers and overall service delivery structure differences between Ethiopia and European countries. Also, the study finding was not consistent with the study conducted in Malaysia which found that more than half of the respondents were satisfied with the service that they received 56.7% [32]. This variation may be due to thedifference in the level of health facilities.

In this study, it was found that younger age groups (below 34 years) tend to get high satisfaction than older ages; this is also true after adjusting for the other variables included in the model (multivariate analysis). Also, a significant association was shown in the finding which is in congruence with a study done in Addis Ababa; the study revealed that age is negatively and significantly associated with satisfaction [30]. Because the elders were more experienced than their younger counterparts, they may compare the service with the previous one they encountered.

It was determined that clients who belonged to “illiterate and primary” education levels were more satisfied than the secondary and higher groups, and the association was also significant in multivariate analysis; the finding is similar to several studies, for example, a study conducted by Javed and Hasen [11, 18] and Elsaba [19]. In this study, respondents who travel more than 30 minutes were two times more satisfied than those whose travel time is less than 30 minutes. This is a similar study conducted in Pakistan, and another study in Adama revealed that those who live far (30 minutes of travel time) from the health facility were highly dissatisfied than those who live near [35, 36]. It might be that those who live near the health institution had the chance to use the facility any time they want which makes them more satisfied.

Also, the findings of this study revealed that clients respected by health care providers were significantly associated with satisfaction. Those who reported that they were respected by the care provider were two times more likely satisfied than those not respected by care providers. This finding is similar to a previous study conducted in Addis Ababa in which the study revealed a significant relationship between the level of satisfaction and client respect, and clients who were respected by the health care service provider were more satisfied compared to those not respected [21].

In this study, client satisfaction with the ANC services was significantly related to privacy during the ANC session. Study subjects who feel secured regarding their privacy were more satisfied than their referents. This finding is in congruence with a study done in Bahir Dar in which respondents whose privacy was maintained were about two times more likely to be satisfied than those whose privacy was not maintained [22]. Findings of this study also showed that cleanness of the facility had a significant association with satisfaction. This is in line with a study conducted in Wolaita where respondents who reported that the institution was clean were seven times as high to be satisfied than those who reported that the institution was not clean [23]. It is obvious that a clean and attractive environment will enhance someone's satisfaction with the existing service.

## 6. Conclusions

Generally, around three-fourths of the respondents were satisfied with the service that they received. Most of the clients were satisfied with privacy, approaches of care from the care provider, interpersonal and technical aspects, facility-related factors, and cleanness of the center. Relatively low satisfaction was reported on the communication and information domain. Age, education status, distance from the facility, respect of privacy, cleanness of the facility, and provider respectfulness were the significant predictors of the satisfaction level. The authors of this study kindly request any additional support from governmental and nongovernmental organizations at the national and international levels including family doctor's practice to improve maternal care quality more specifically antenatal care services.

## Figures and Tables

**Table 1:** Sociodemographic characteristics of respondents in Hossana town public health centers, Southern Ethiopia (*n* = 303).

Sociodemographic characteristics	*f*	%
Age (*N* = 303)		
<24	122	40.3
25-29	113	37.3
30-34	46	15.2
≥35	22	7.3
Median	25	
Marital status		
Currently married	297	98.0
Currently unmarried	6	2
Educational status		
Illiterate	35	11.6
Primary school	85	28.1
Secondary school	104	34.3
Tertiary school	79	26.1
Religion		
Orthodox	66	21.8
Protestant	196	64.7
Muslim	28	9.2
Catholic	13	4.3
Ethnicity		
Hadiya	209	69.0
Amhara	26	8.6
Silte	23	7.6
Gurage	19	6.3
Kembata	22	7.3
Oromo	4	1.3
Occupation		
Currently unemployed	170	56.1
Currently employed	133	43.9
Residence		
Urban	268	88.4
Rural	35	11.6
Health facility		
NEMH	79	26.1
Hossana HC	116	38.3
Lichamba HC	39	12.9
Bobuco HC	69	22.8

**Table 2:** Level of client satisfaction with different domains of satisfaction in Hossana town, public health centers, Southern Ethiopia (*n* = 303).

Components	Level of satisfaction (*n* = 303)	
	Satisfied *N* (%)	Not satisfied *N*
Approaches of care	243 (80.2)	60 (19.8)
Interpersonal and technical aspects	242 (79.9)	61 (20.1)
Communication and information	228 (75.2)	75 (24.8)
Privacy	277 (91.4)	26 (8.6)
Physical facility	251 (82.8)	52 (17.2)
Cleanness	265 (87.5)	38 (12.5)
Overall satisfaction	226 (74.6)	77 (25.4)

**Table 3:** Multivariate analysis showing factors associated with maternal satisfaction with antenatal care in public health centers, Hosanna town, Southern Ethiopia (*n* = 303).

Variables	Satisfied *N* (%)	COR (95% CI)	AOR (95% CI)
Age			
≤24	94 (77%)	3.3 (1, 8.5)	5.584 (1.76, 17.7)
25-29	89 (78%)	3.7 (1.4, 9.5)	8.6 (2.5, 28)
30-34	32 (69.5%)	2.2 (0.8, 6.5)	2.6 (0.757, 9.287)
≥35	11 (50%)	1	1
Educational status			
Illiterate	30 (85.7%)	2.4 (0.8, 7.1)	4.53 (1.228, 16.751)
Primary school	75 (88%)	3.1 (1.4, 7.0)	3.082 (1.169, 8.124)
Secondary school	65 (64.3%)	0.6 (0.3, 1.2)	0.538 (0.247, 1.17)
Tertiary school	56 (73.6%)	1	1
Residence			
Urban	205 (76.5%)	2.2 (1.0, 4.5)	1.987 (0.785, 5.0)
Rural	1 (0.4%)	1	1
Wanted status of pregnancy			
Yes	209 (76%)	2.05 (0.914, 4.59)	1.977 (0.671, 5.823)
No	17 (60.7%)	1	1
Parity			
Primipara	81 (78.6%)	1.397 (0.79, 2.45)	1.112 (0.511, 2.422)
Multipara	145 (72.5%)	1	1
Privacy respected			
Yes	214 (80.4%)	8.5 (4, 18)	8 (3.375, 19.263)
No	12 (32%)	1	1
Care provider was respectful			
Yes	207 (77.8%)	3.3 (1.6, 6.7)	2.578 (1.249, 5.325)
No	19 (51%)	1	1
Institution clean			
Yes	182 (77%)	1.87 (1, 3.35)	3.596 (1.424, 9.079)
No	44 (64.7%)	1	1
Distance from home			
≤30 minutes	166 (79.8%)	2.306 (1.348, 3.945)	2.804 (1.423, 5.522)
>30 minutes	60 (63.2%)	1	1
Waiting time			
≤30 minutes	198 (77.6%)	2.481 (1.302, 4.729)	2.17 (0.967, 4.878)
>30 minutes	28 (58%)	1	1
Expectation		1.067 (0.98, 1.161)	1.118 (0.986, 1.269)
Knowledge		1.130 (1.021, 1.25)	1.07 (0.956, 1.198)

## Data Availability

The data supporting the findings of this work is available at the hands of the corresponding author.
